# Traditional knowledge on zootherapeutic uses by the Saharia tribe of Rajasthan, India

**DOI:** 10.1186/1746-4269-3-25

**Published:** 2007-06-05

**Authors:** Madan Mohan Mahawar, DP Jaroli

**Affiliations:** 1 Department of Zoology, Government Post Graduate College, Sawai Madhopur, Rajasthan, India; 2 Department of Zoology, University of Rajasthan, Jaipur, Rajasthan, India

## Abstract

The present zootherapeutic study describes the traditional knowledge related to the use of different animals and animal-derived products as medicines by the Saharia tribe reside in the *Shahabad *and *Kishanganj Panchayat Samiti's *of *Baran *district of Rajasthan, India. A field survey was conducted from April to June 2006 by performing interview through structured questionnaire with 21 selected respondents, who provided information regarding use of animals and their products in folk medicine. A total of 15 animal species were recorded and they are used for different ethnomedical purposes, including cough, asthma, tuberculosis, paralysis, earache, herpes, weakness, muscular pain etc. The zootherapeutic knowledge was mostly based on domestic animals, but some protected species like the peacock (*Pavo cristatus*,), hard shelled turtle (*Kachuga tentoria*), sambhar (*Cervus unicolor*) were also mentioned as medicinal resources. We would suggest that this kind of neglected traditional knowledge should be included into the strategies of conservation and management of faunistic resources. Further studies are required for experimental validation to confirm the presence of bioactive compounds in these traditional remedies and also to emphasize more sustainable use of these resources.

## Background

### Zootherapy and its importance

The healing of human ailments by using therapeutics based on medicines obtained from animals or ultimately derived from them is known as zootherapy [1]. In modern society, zootherapy constitutes an important alternative among many other known therapies practiced worldwide [2]. Research interest and activities in the areas of ethnobiology and ethnomedicine have increased tremendously in the last decade. Since the inception of the disciplines, scientific research in ethnobiology and ethnomedicine has made important contributions to understanding traditional subsistence and medical knowledge and practice [3]. Since ancient time animals, their parts and their products have constituted part of the inventory of medicinal substances used in various cultures. This phenomenon is marked by both a broad geographical distribution and very deep historical origins [4]. In Pakistan 31 substances were listed (animal parts and products), constituting 9% of all the medicinal substances in the inventory of traditional medicines [5]. A survey of traditional materia medica in use in the markets of Israel recorded 20 substances of animal origin [6]. Alves and Rosa carried out a survey in fishing communities located in the North and Northeastern regions of Brazil and recorded 138 animal species was used as medicine [7]. Costa Neto describes the use of 180 animal species as medicinal resources in the state of Bahia, Northeastern Brazil [8]. In a review Alves and Rosa discusses ecological, cultural (traditional knowledge), economical, and sanitary aspects of zootherapy and describes many reasons to why studies on the use of animals, integrally or in parts, as medicines and their implications should be carried out and recorded [2].

In India, since times immemorial, great work was done in this field and documented in works like *Ayurveda *and *charaka Samhita*. Additionally immense knowledge has come down to modern times through folklore as various practices became a part of tradition amongst various groups. We can find that in our rural people still use various animal products and by-products for cure of various diseases. For example, honey is used as expectorant, cattle urine has been used as a therapeutic. All this knowledge has once again come to the limelight, as there has been a sort of disillusionment with the current allopathic cure, as it has got its own side effect and in fact has no cure for various diseases. Therefore people are looking for traditional remedies for the treatment of ailments. But in India this traditional knowledge is fast eroding due to modernization. Thus there is an urgent need to inventorise and record all ethnobiological information among the different ethnic communities before the traditional cultures are completely lost [9]. The studies on the therapeutic uses of animals and their body parts have been neglected, when compared to plants [10]. This paper deals with the zootherapeutic aspects of the Saharia tribe of Rajasthan, India to narrow the gap of our knowledge in this field.

### Traditional zootherapeutic uses in India

In India, nearly 15–20 percent of the Ayurvedic medicine is based on animal-derived substances [11]. The Hindu religion has used five products (milk, urine, dung, curd and ghee) of the cow for purification since ancient times [12]. Different ethnic groups use animal-derived substances for healing human ailments in present times in India. Ghosh and Maiti identified 20 species of mammals have been proved as vital sources of tribal medicine [13]. Dutta et al studied use of certain animals and their product in medical treatment by tribal people in Assam. [14]. S.K. Sharma describes use of birds and animals to cure ailments of human beings and domestic cattle by Bhil tribe of Rajasthan. [15]. Jamir and Lal describe the traditional method of treating various kinds of ailments using twenty six animal species and their products by different Naga tribes [16]. Patil found that the tribals of Nandurbar district (Maharashtra) have been use wild animal parts as medicines along with plants. This study assesses 15 species of animals used by the tribals like Bhils, Gamits, Koknas and Pawaras as medicine [17]. Ranjit Singh et al describe the Ethno-entomological practices in Tirunelveli district, Tamil Nadu. In this investigation, 11 species of insects used to prepare traditional medicine [18]. Banerjee et al describe that Honey, as a product from bees, has multiple properties, and is being therapeutically used since time immemorial. It's antibacterial, anti-inflammatory and wound healing properties are promising [19]. Gupta et al describe the traditional knowledge of local communities in district Kachchh and listed about 34 animals and bird species, which are used in primary health care of human beings and livestock [20]. Kalita et al study the plant and animal based folk medicine used by people of Dibrugarh district, Assam for treatment of eleven different diseases. In this study, information on utility of 19 plant species and 4 animal species is collected [21]. Solavan A et al carried out a study among nine tribes spread over four districts of Tamil Nadu, India and identified the traditional therapeutic uses of sixteen different animal's species, consisting of mammals (6), birds (5), reptiles (2), arthropods (2) and annelid (1), for the treatment of over 17 kinds of ailments [10]. Mahawar and Jaroli carried out a study among the inhabitants, whose are living surrounding the Ranthambhore National Park, India and identified a total of 15 animals were used comprising 20 therapeutic purposes [22]. The Chakhesang tribe of Nagaland also uses twelve mammals, one bird, one reptile, two amphibians, one fish, one mollusk, one annelid and four arthropods for treatment of various ailments [23]. Kakati and Doulo studied Ao tribe of Nagaland and identified twenty five different vertebrate species for traditional therapeutic use, of which, some have become rare [24].

### Saharia tribe and study area

**Figure 1:**
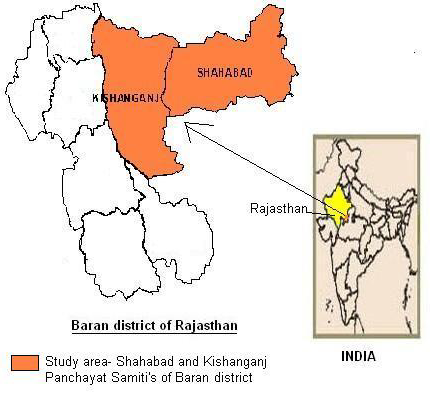
Map of study area.

Sahariya, the only primitive tribe of the Rajasthan state, resides in the *Shahabad *and *Kishanganj Panchayat Samiti's *of *Baran *district (24–25' to 25–25' North latitudes, 76–12' to 77–26' east longitudes and 262 mts. Altitude) (Figure 1). The total population of Saharia is 79,312 with sex ratio of 951 females per 1000 males. A majority (93%) of the Saharia population inhabits of Kishanganj and Shahbad. The major occupation of the head of the households is either agricultural or other labour (82%), followed by cultivation (14.3%), service (1.6%) and business (1.3%) [25]. Ox, Cow, Buffalo, Sheep, Goat are major domesticated animals used in agriculture by them. They are non-vegetarians and eat the flesh of goat, sheep and birds. Nearly half of the households were living at a distance of > 10 km from primary health center for allopathic treatment [25]. The district has a dry climate except in the monsoon seasons and average rainfall is 854.5 mm. The forest covers an area of 2.15 lacs hectare of the district. The main wild animals found in this area are Panther, spotted deer, Wild Bear, Chinkara, Sambhar, Langoor, Jackal etc. Birds found in the district are Bulbul, Sparrow, Peacock, Saras, and Partridges etc. Among the poisonous snakes Cobra and Viper are common. The Sahariya people maintained ecological equilibrium with their environment for ages, despite low level of technology. Sahariya live in infrastructural weak and remote areas, not well connected through road/bridge network even now. Lack of exposure to modern life and historic exploitation by landlords who paid them fewer wage, has left Sahariyas economically weak tribe. Trivedi has provided detailed information regarding the use of medicinal plants by this tribe in a major research project on ethnomedicinal plants of Rajasthan state [26]. However animals and minerals which are being put to therapeutic use in this tribe are yet to be highlighted. So we have taken up the zootherapeutic aspects of Saharia tribe in this paper.

## Methodology

**Figure 2:**
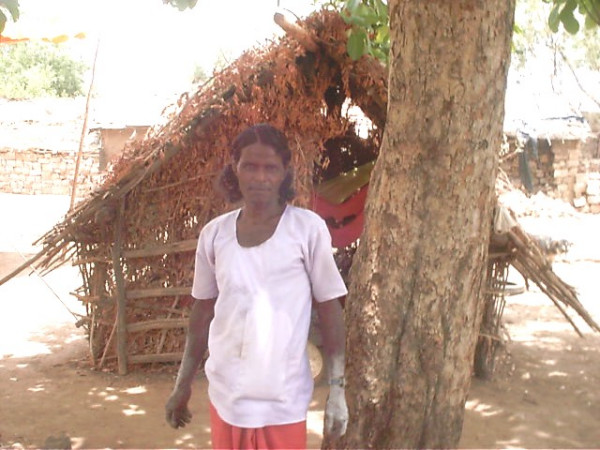
A Saharia man.

**Figure 3:**
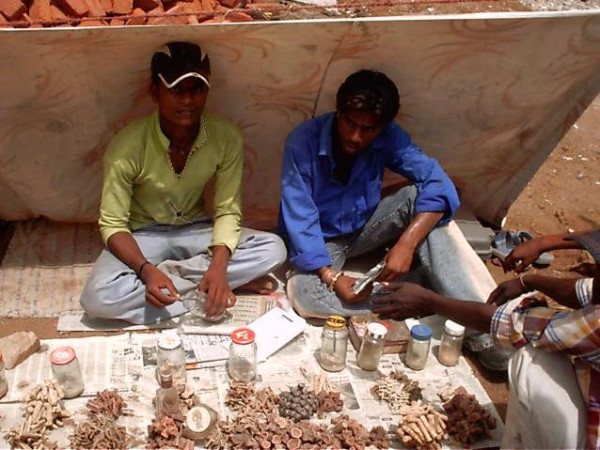
Local vendors selling medicines at a fair.

A field survey was conducted from April to June 2006 by performing interview through structured questionnaire with 21 selected respondents (17 men and 4 women), to collect information about traditional knowledge regarding use of animals and their products in folk medicine (Figure 2, 3). These respondents were local herbalists, healers, farmers, and midwives and between 30–64 age groups. The selection of respondents was based on their recognition as knowledgeable members concerning folk medicine. Prior consent was taken from the respondents for recording of the information. We ask the respondents whether they know the use of animals in the healing practices. Mostly they have knowledge on plant based medicine but they also know some use of animals in therapeutics. We questioned them about the animal remedies and which of them is prescribed for which ailment. We also ask the modes of preparation of remedies and how the medicines are administered, since this kind of information indicates how a given medicine can be therapeutically efficient in terms of the right ingredients and the proper dose. According to them, their knowledge of folk medicine was acquired mainly through parental heritage, or because they have experience about medicinal value of animals to heal their kin or themselves. The scientific name and species of animals were identified by using relevant and standard literature [27,28].

For the data analysis, fidelity level (FL) calculated that demonstrates the percentage of respondents claiming the use of a certain animal for the same major purpose, was calculated for the most frequently reported diseases or ailments as:

FL (%) = *N*p × 100/*N*

Where *N*p is the number of respondents that claim a use of a species to treat a particular disease, and *N *is the number of respondents that use the animals as a medicine to treat any given disease [29].

## Result and discussion

Data obtained from field surveys are summarized in Table 1. The *respondents *have been provided all the information regarding local name of the animal, part or product used to cure which ailment and method of preparation. In this study, we identified 15 animal species, which are being used for 19 medicinal purposes. These animals are used as whole or body part or byproduct like milk, blood, organ, flesh, antler, feather etc. for the treatment of different kind of human ailments including cough, asthma, tuberculosis, paralysis, earache, herpes, weakness, muscular pain etc [Table 1].

**Table 1:** Medicinal uses of animals and animal parts in traditional therapy By Saharia tribe in district Baran (Rajasthan).

English Name	Scientific Name	Local Name	No. of Respondents mentions	Fidelity level (FL)	Part used	Medicinal use	Method of preparation	Related earlier reported use in India [References]
1. Bivalves	*Mactra sp*.	Seepi	11	52%	Ash of shell	Weakness	Ash of shell is taken for weakness.	Shell use for acne by Mogya, Bawaria, Meena tribe of Rajasthan. [22]
2. Camel	*Camelus dromedaries*	Uant	15	71%	Milk	Muscular pain	Used as massage cream in muscular pain.	Dung use for constipation in Kachchh. [20]
3. Crab	*Cancer pararus*	Kekada	6	29%	Whole body	Cough, asthma, T. B.	Ash of crab is used in lung diseases as cough, asthma, T. B. etc.	Whole body used for Jaundice and other liver disorders by tribes of Nagaland. [16]
4. Labeo	*Labeo rohia*	Machchhi	10	48%	Cervical vertebrae	Urine Problem	A fish cervical vertebra is rubbed with water and this essenced water is taken in urine blockage problem.	
5. Goat	*Capra indicus*	Bakri	12	57%	Urine	Cough, tuberculosis	Urine of goat administered orally to cure tuberculosis.	Also reported by Ao tribe for asthma, T.B., paralysis [16] [24] and for insect bite by Tamilnadu tribe [10] and by Mogya in paralysis. [22]
6. Goat	*Capra indicus*	Bakri	21	100%	Bones of Legs	Weakness	Soup of leg's bone used to cure weakness.	Same use reported in Kachchh. [20]
7. Hardshelled Turtle.	*Kachuga tentoria*	Kachhua	6	29%	Carapace	Burn	Ash of carapace mix with coconut oil and use for skin burns.	
8. Hardshelled Turtle.	*Kachuga tentoria*	Kachhua	5	24%	Carapace Flesh	Cough, asthma, T. B	Ash of carapace is used in lung diseases as cough, asthma, T. B. etc.	Same use by Mogya, Bawaria, Meena tribe of Rajasthan [22] and Ash of *Lissemys punctatus*' Carapace is used for healing of internal injuries, prurities and cough (Kachchh). [20]
9. Honey bee	*Apis indica*	Mokh	17	81%	Honey	Eye disease	Used as eye drops to cure eye disease.	Same use by Mogya, Bawaria, Meena tribe of Rajasthan [22] Honey is used for cough and could and asthma (Tamilnadu tribes). [10] [18]
10. Horse	*Equus sp*.	Ghoda	2	09%	Semen	Tetanus, Rabies	Administered orally to cure.	
11. Human	*Homo sapiens*	Manakh	3	14%	Bones	Herpes	Bone is grounded with water and this paste is applied in Herpes.	
12. Human	*Homo sapiens*	Manakh	19	90%	Urine	Wound	Human urine is used as antiseptic for wound healing.	Also reported by Naga tribe of Nagaland and Mogya, Bawaria, Meena tribe of Rajasthan. [16][22]
13. Indian Peafowl	*Pavo cristatus*	Mor	6	29%	Leg	Ear infections	Peacock's leg is rubbed with water and this essenced water is used in ear infections	Also reported in Naga tribe of Nagaland [16][24] and Bhil [15], Mogya, Bawaria of Rajasthan [22] Legs boil with oil in kachchh [20] and Maharastra. [17]
14. Indian Peafowl	*Pavo cristatus*	Mor	4	19%	Feather	Infertility	Rounded spots of feather mix with Jaggery.	The ash of feather is used for cough in Maharastra [17] and feather used in hiccups by the Tamilnadu tribes. [10]
15. Pigeon	*Columba livia*	Kabutar	14	67%	Fresh blood, meat, Feather	Paralysis	The fresh blood is massaged externally to treat paralysis. Soup of meat and feather is useful in paralysis.	Same use reported in Bhil [15] Mogya, Bawaria of Rajasthan [22] Kachchh [20] and Tamilnadu. [10] Blood is also use for epilepsy in tamilnadu [10] and Flesh use for asthma and weakness by Naga tribes. [16] [24]
16. Prawn	*Macrobrachium malcolmsonii*	Jhinga machchi	2	09%	Dried powder	Tuberculosis	Taken for cure of Tuberculosis.	
17. Sambhar	*Cervus unicolor*	Sambhar	3	14%	Antler	Herpes	Antler is rubbed with water this paste is applied in Herpes.	Antler use in eye ailments by Mogya, Bawaria, Meena tribe of Rajasthan [22] and Kachchh [20]
18. Sheep	*Capra sp*.	Menda	16	76%	Milk	Muscular pain	Used as massage cream in muscular pain.	Same use by Mogya, Bawaria, Meena tribe of Rajasthan [22]
19. Snail	*Pila sp*.	Sankh	11	52%	Ash of shell	Weakness	Ash of shell is taken for weakness.	Flesh is use for asthma, tuberculosis, stomach disorders and eye related problems by tribes of Nagaland. [16]

Fidelity level (FL) demonstrates the percentage of respondents claiming the use of a certain animal for the same major purpose. The uses of animals that are commonly known by the respondents have higher fidelity level than less common known. The soup of *Capra*' legs bone used to cure weakness has the highest FL (100%) and semen of *Equus sp*. has the lowest (9%). Obviously, the remedies for frequently reported aliments have the highest FL value and those with low number of reports have lowest FL values.

The relevance of highlighting the use of a number of animal-based drugs to treat various diseases by different ethnic communities of India has been established by previous authors, in different landscapes. Gupta Leena et al inventoried 34 animal species used as remedies in the kachchh of Gujarat [20], Solvan A et al reported the use of 26 animal species by *Kanikar, Paliyar *tribe of Taminadu [10], Jamir N S et al reported the use of 26 species by the *Naga *tribe of Nagaland [16], Kakati L N et al identified 25 species used by *Ao *tribe of Nagaland [24]and Mahawar and Jaroli reported the use of 15 species by the *Mogya, Meena, Bawaria *of Rajasthan [22].

Different animals used for healing by the Saharia are also being used by various groups in India. Some animals or their products are being put to similar uses, such as the urine of *Capra indicus *for asthma, T.B., paralysis is also used by Ao and Naga tribes of Nagaland and Mogya, Bawaria of Rajasthan [16,22,23] and the Soup of leg's bone used to cure weakness is also reported in Kachchh region [20]. The ash of *Kachuga tentoria *carapace is used in cough, asthma, T. B. etc. also reported By Mogya of Rajasthan [22]. *Homo sapiens *urine used as antiseptic for wound healing is also reported by Naga tribe of Nagaland and Mogya, Bawaria, Meena tribe of Rajasthan [16,22]. *Pavo cristatus *legs used for ear infection is also similar in tribes of Nagaland and Rajasthan [15,16,22,24] but Legs are boil with oil in Maharastra and kachchh for similar purpose [17,20]. Fresh blood of *Columba livia *is used for paralysis is also reported by other groups of Rajasthan [15,22] Kachchh [20] and Tamilnadu [10]. Antler of *Cervus unicolor *used for eye ailments in Rajasthan [22] and Kachchh region [20]. The *Capra sp*. milk is used for muscular pain is also reported for same purpose by Mogya, Bawaria and Meena of Rajasthan [22].

However some of these animals and their products are being used for the treatment for other diseases in different parts of India, such as the ash of crab is used in lung diseases as cough, asthma, T. B. etc. in this area but the whole body used for Jaundice and other liver disorders by tribes of Nagaland [16]. The *Pavo cristatus' *feather uses in infertility in this region but the ash of feather is used for cough in Maharastra [17] and in hiccups by the Tamilnadu tribes [10]. Fresh blood of *Columba livia *is used for paralysis by this tribe but it has been reported for epilepsy in Tamilnadu [10] and the flesh use for asthma and weakness by Naga tribes [16,24]. *Lissemys punctatus *carapace is used for healing of internal injuries and cough in Kachchh region [20]. Flesh of *Pila sp *is use for asthma, tuberculosis, stomach disorders and eye related problems by tribes of Nagaland. [16]

The *Homo sapiens *bones for herpes, Cervical vertebrae of *Labeo rohia *for Urine Problem, *Camelus dromedaries *milk for muscular pain, the ash of *Lissemys punctatus *carapace for burn, *Macrobrachium *for tuberculosis, semen of *Equus sp. for *tetanus and rabies, antler of *Cervus unicolor *for herpes and the shell of *Mactra sp*. and *Pila sp*. for weakness are used by Saharia in this region has not possibly been reported earlier in India.

Some animals are also being reported in other parts of the world, such as shell of bivalve used in Brazil to treat weakness [7], Honey bee used in Brazil and Sudan for a wide variety of ailments, such as cough, liver disorders and gastrointestinal disorders [7,30]; Flesh of *Marcrobrachium *used in Brazil to treat irritation when milk teeth are erupting [7].

The Saharias used some protected species like the *Pavo cristatus*, hard shelled turtle (*Kachuga tentoria*), sambhar (*Cervus unicolor*) are also included as medicinal resources. We would suggest that this kind of neglected traditional knowledge should be included into the strategies of conservation and management of faunistic resources. Alves and Rosa suggest numerous reasons to urgently re-think the medicinal use of animal products in traditional medicine both in humans and animals. For doing this, the rarity of species, the unnecessary suffering involved in the harvesting (e.g., hunting, fishing) process, and the possible health risks linked to the administration of the animal-based remedies [2]. Further studies are required for experimental validation to confirm the presence of any bioactive compounds in these traditional remedies and also to emphasize more sustainable use of these resources.

## Conclusion

The result of our survey among Saharia people revealed the use of 15 animal species for 19 medicinal purposes. We would suggest for further studies on these traditional remedies to confirm the presence of any bioactive compounds and also include this traditional knowledge into the strategies of conservation and management of faunistic resources for sustainable use.
